# Supplementary material for: Detection of CADM1, MAL, and PAX1 Methylation by ddPCR for Triage of HPV-Positive Cervical Lesions
Source: Biomedicines. 2025 Jun 12;13(6):1450. doi: 10.3390/biomedicines13061450 (PMC12190569; doi:10.3390/biomedicines13061450)
Supplement: Supplementary file 1 [file biomedicines-13-01450-s001.zip › Supplementary Material.pdf]

Supplementary Materials

Table S2. Sequences of oligonucleotide primers and probes used in methylation-specific qPCR and ddPCR assays.

Target Gene	Oligonucleotide	Sequence
CADM1	Forward primer	5′-ATT TTA TTA GTT GTT GGT TCG GGT-3′
	Reverse primer	5′-CTC GAC AAC ACT ACT CGC C-3′
	Detection type probe	5′-FAM-ACC TAC CTC AAA CTA ACG ACG TTA ACT ACC TCC GA-BHQ1-3′
MAL	Forward primer	5′-GCG TAG TAT TAA GTA GAG AGG TTC G-3′
	Reverse primer	5′-AAT AAA AAA TAA AAC CGA CCG C-3′
	Detection type probe	5′-FAM-ACT AAA CCG ACG CTA ATT CGA CGA CGC T-BHQ1-3′
PAX1	Forward primer	5′-TCG TTA GGG AGA AAG GAA TTT GT-3′
	Reverse primer	5′-TAA ATC CGA CGC CCT CCT A-3′
	Detection type probe	5′-FAM-TTT CGT CGG TCG CGT TTG GG-BHQ1-3′
ACTB (control)	Forward primer	5′-TGG TGA TGG AGG AGG TTT AGT AAG T-3′
	Reverse primer	5′-AAC CAA TAA AAC CTA CTC CTC CCT TAA-3′
	Detection type probe	5′-HEX-ACC ACC ACC CAA CAC ACA ATA ACA AAC ACA-BHQ1-3′
ACTB (non-converted)*	Forward primer	5′-GCG CCG TTC CGA AAG TT-3′
	Reverse primer	5′-CGG CGG ATC GGC AAA-3′
	Detection type probe	5′-HEX-ACC GCC GAG ACC GCG TC-BHQ1-3′

HEX, Hexachlorofluorescein; FAM, 6-Carboxyfluorescein; BHQ1, Black Hole Quencher 1. *qPCR conditions were the same as for *ACTB* (see Section 2.4 of the manuscript).

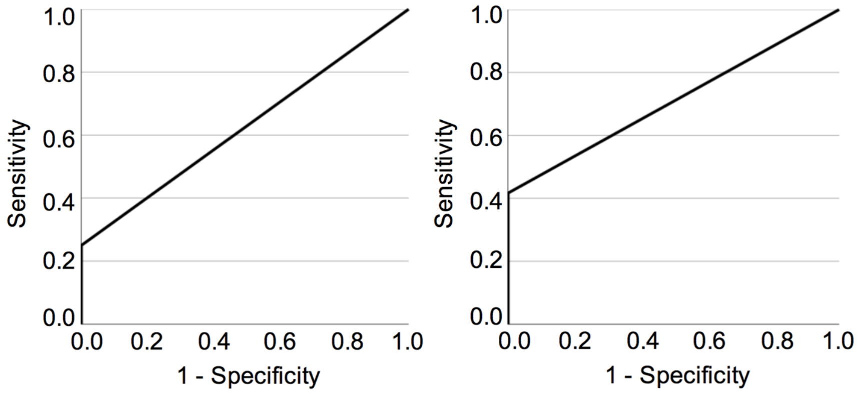

(a) **(b)**

Figure S1. ROC curve analysis based on combined *CADM1* and *MAL* methylation markers. **(a)** NILM (HPV+)+LSIL vs HSIL+Carcinoma. **(b)** NILM (HPV+) vs Carcinoma.

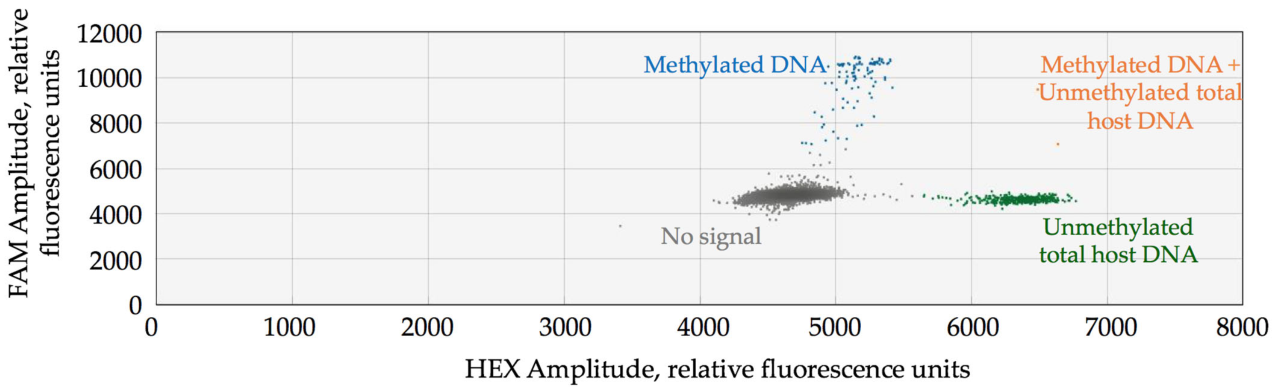
FAM Amplitude, relative fluorescence units
12000
10000
8000
6000
4000
2000
0
0
1000
2000
3000
4000
5000
6000
7000
8000
HEX Amplitude, relative fluorescence units
Methylated DNA
Methylated DNA + Unmethylated total host DNA
No signal
Unmethylated total host DNA

(a)

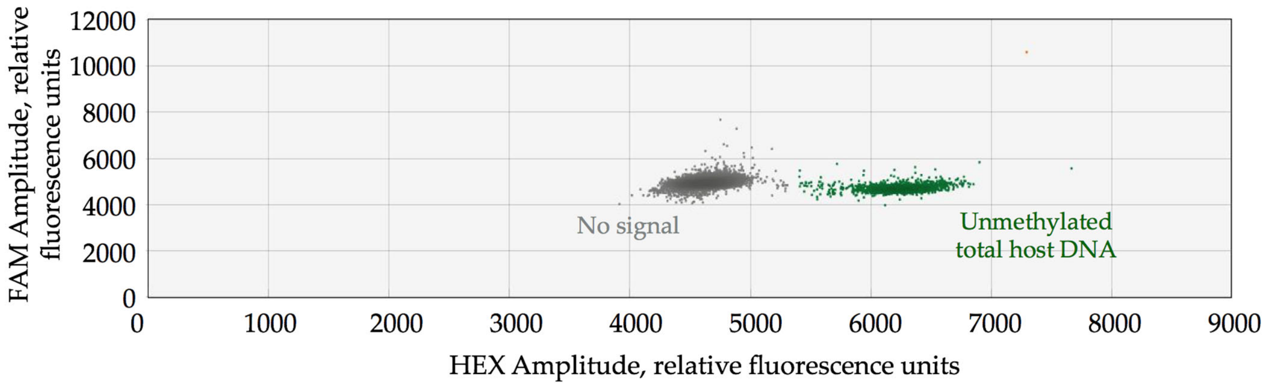
FAM Amplitude, relative fluorescence units
12000
10000
8000
6000
4000
2000
0
0
1000
2000
3000
4000
5000
6000
7000
8000
9000
HEX Amplitude, relative fluorescence units
No signal
Unmethylated total host DNA

(b)

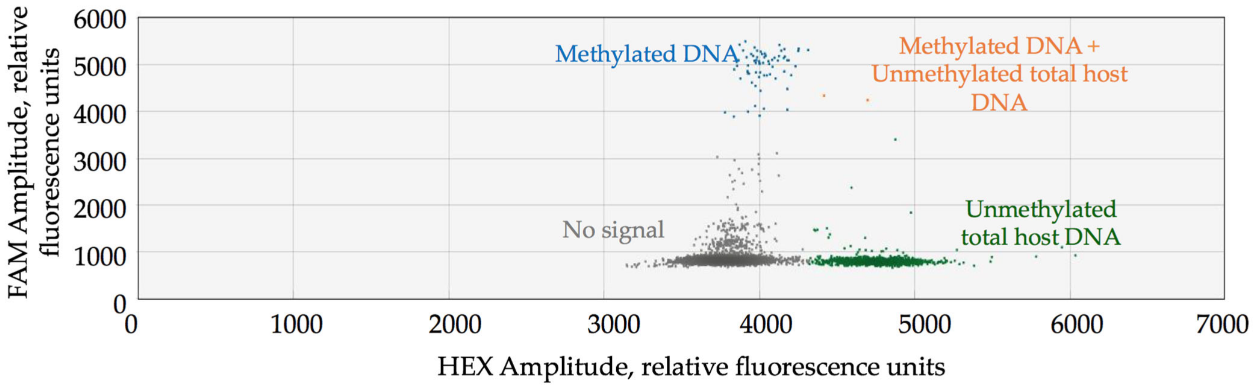
FAM Amplitude, relative fluorescence units
6000
5000
4000
3000
2000
1000
0
0
1000
2000
3000
4000
5000
6000
7000
HEX Amplitude, relative fluorescence units
Methylated DNA
Methylated DNA + Unmethylated total host DNA
No signal
Unmethylated total host DNA

(c)

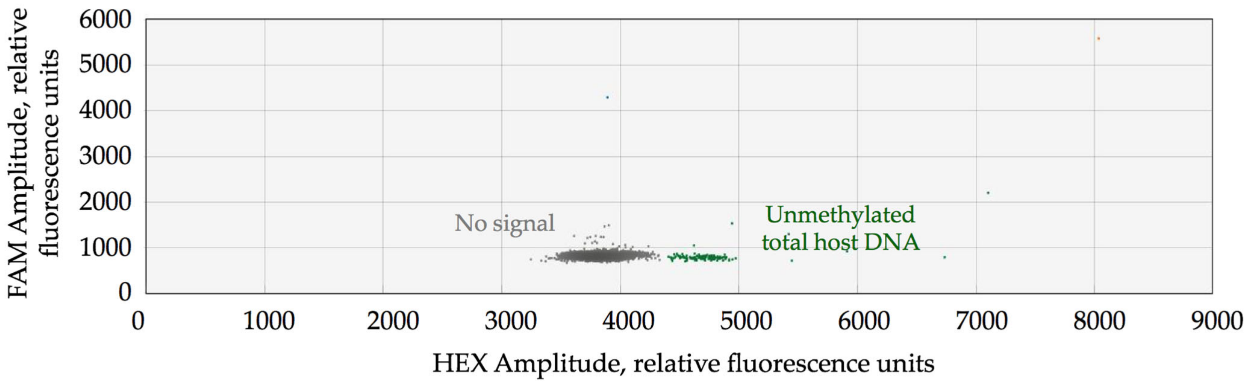
FAM Amplitude, relative fluorescence units
6000
5000
4000
3000
2000
1000
0
0
1000
2000
3000
4000
5000
6000
7000
8000
9000
HEX Amplitude, relative fluorescence units
No signal
Unmethylated total host DNA

(d)

Figure S2. Representative 2D fluorescence amplitude plots of ddPCR results for the *MAL* and *PAX1* genes. Probes specific for methylated *MAL* and *PAX1* DNA were labeled with FAM, whereas probes for unmethylated total host DNA (*ACTB*) were labeled with HEX. **(a)** A positive sample showing clusters corresponding to methylated *MAL* DNA (FAM), unmethylated *ACTB* DNA (HEX), and double-positive droplets (FAM + HEX). **(b)** A negative sample showing only unmethylated *MAL*/*ACTB* DNA, with a few false-positive droplets in the upper right quadrant. **(c)** A positive sample showing clusters corresponding to methylated *PAX1* DNA (FAM), unmethylated *ACTB* DNA (HEX), and double-positive droplets (FAM + HEX). **(d)** A negative sample showing only unmethylated *PAX1*/*ACTB* DNA, with a few false-positive droplets in the upper right quadrant.

Table S3. Sensitivity and specificity of *CADM1*, *MAL*, *PAX1*, and the combined *CADM1* + *MAL* methylation markers for distinguishing HSIL+ (cytological or histological) from NILM/LSIL in HPV-positive women.

Category	*CADM1*	*MAL*	*PAX1*	*CADM1+MAL*
AUC	0.671	0.643	0.610	0.776
Sensitivity, (%)	47.1	47.2	58.8	42.9
Specificity, (%)	88.1	83.3	71.4	100
Cut-off, (%)	0.09	0.01	1.67	2.785

Data are presented as area under the curve (AUC), sensitivity, specificity, and optimal cut-off values from ROC curve analysis.

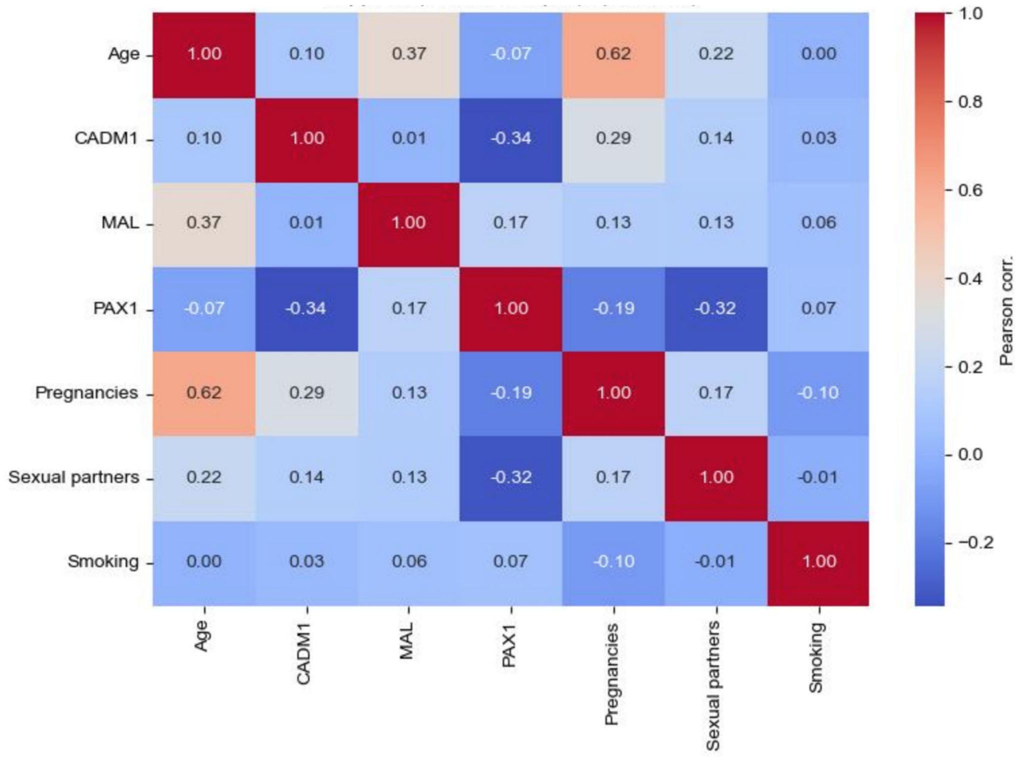

Figure S3. Correlation matrix (Pearson's r) between methylation markers (*CADM1*, *MAL*, *PAX1*) and clinical-demographic variables (age, parity, smoking, number of sexual partners) in the study cohort.

Table S4. Sensitivity and specificity of *CADM1, MAL, PAX1*, and the combined *CADM1 + MAL* methylation markers for cervical cancer detection in HPV-positive women.

**Category**	***CADM1***	***MAL***	***PAX1***	***CADM1+MAL***
AUC	0.777	0.770	0.813	0.912
Sensitivity, (%)	50	62.5	87.5	70
Specificity, (%)	100	96.9	68.7	100
Cut-off, (%)	4.57	0.085	1.745	2.785

Data are presented as area under the curve (AUC), sensitivity, specificity, and optimal cut-off values from ROC curve analysis.
